# Prevalence of *Trypanosoma cruzi* infection in active military population of The Colombian National Army gathered in five departments

**DOI:** 10.1371/journal.pone.0223611

**Published:** 2019-10-09

**Authors:** Claudia Méndez, Maria Clara Duque, Yanira Romero, Julie Pérez, Omaira Rodríguez, Camilo A. Correa-Cárdenas, Maria Teresa Alvarado, Omar Cantillo-Barraza

**Affiliations:** Grupo de Investigación en Enfermedades Tropicales del Ejército (GINETEJ), Laboratorio de Referencia e Investigación, Dirección de Sanidad, Bogotá, Colombia; Faculty of Science, Ain Shams University (ASU), EGYPT

## Abstract

**Background:**

The National Army of Colombia is present in all of the national territory, focused in sylvatic zones where they are exposed continually to potential risk of transmission of *Trypanosoma cruzi*, the etiological agent of the Chagas disease. People of this study were active personal that were born and lived during their first years in endemic areas of transmission through domiciled vectors as *Rhodnius prolixus*.

**Aim:**

The main aim of this study was to estimate the prevalence of Chagas disease in the active military population of the National Army, gathered in five departments.

**Methods:**

An observational and descriptive study with cross-sectional analysis was carried out. Blood sample each patient in order to apply serological diagnosis by two different Enzyme Linked ImmunoSorbent Assay tests, following the algorithm of National Institute of Health, Colombia. In cases of serum results with inconsistencies, a Hemagglutination Inhibition test and Indirect Immunofluorescence assay test were performed to solve inconclusiveness. Positive samples by two different tests were considered seropositive. Additionally, to each positive sample by at least one serological test, we did extraction of DNA for molecular diagnosis.

**Results:**

295 serums were analyzed and two of them were positive in order to get a prevalence of 0.68%. Two samples analyzed by molecular diagnosis were negative.

**Conclusion:**

The prevalence was < 1% It is probable the infection in the seropositive individuals occurred before enlisting in the military service due to origin locations with transmission such as Casanare and Boyacá. These findings allow defining the prevention and control programs of chronic cases and reduction in the disease burden.

## Introduction

Chagas disease is a zoonosis caused by the protozoan hemoflagellate *Trypanosoma cruzi*, which is transmitted to humans mainly by insects of the triatominae subfamily (Hemiptera: Reduviidae) throughout skin contact and mucous membranes with feces and other secretions of these insects [1]. Other ways of transmission are oral, congenital, blood transfusion, organ transplant and laboratory accidents. Currently, around 8 million people are infected with the parasite and at least 10.000 deaths related to this disease occurred per year. Also, it has been estimated that globally, the annual burden of this disease is 752,000 lost working days/years due premature deaths and 806,170 Disability Adjusted Life Year (DALYS) [2].

Vector transmission of *T*. *cruzi* is the most frequently way of Chagas disease transmission in Colombia. In this country 25 species of triatomines have been reported in 423 municipalities located under 2000mamsl distributed in 31 departments [3–5]. *Rhodnius prolixus* is the main vector species of *T*. *cruzi* in Colombia, its domiciliation in part of the Andean region, eastern plains of Orinoco and the Sierra Nevada of Santa Marta has been responsible for most of the transmission during years [3, 6, 7]. On Chagas disease has been designed and implemented a national program for the interruption of intradomestic transmission by triatomine in the country (www.minsalud.gov.co). The presence inside houses of other species such as *Triatoma dimidiata* in other geographic areas of the country also contributes to domestic transmission *of T*. *cruzi* [8]. In contrast non-domiciled vectors such as *Triatomamaculata*, *Triatomavenosa* and *Panstrongylus geniculatus* have been related to peridomestic transmission in the plains of the Colombian Caribbean and some areas in the Andean region [9–11]. Finally, the enzootic transmission is mediated by sylvatic triatominae such as *R*. *pallescens*, *Eratyrus cuspidatus*, *Panstrongylus rufotuberculatus* and *Psammolestes arthuri*, who participate when people enter into sylvatic transmission due to recreational or work activities as patrolling by active military population of The National Army of Colombia [5, 12–14].

Prevalence studies of Chagas disease in Colombia usually have been focused on population of particular age or conditions living in endemic regions [15, 16]. The evaluation of infection in children under 14 years of age for the estimation of recent infection and the assessment in children under five years of age for the monitoring of transmission interruption by *R*. *prolixus*, are commonly indicators analyzed [6, 17]. In contrast, few studies have been executed in adult population and its focus has been on searching for infection in pregnant women, surveillance in blood banks and researching in outbreaks linked with oral transmission [18–20]. Even though in Colombia there have not been assessed the dimension of infections in adults that lived during their first years of life in municipalities with *T*. *cruzi* transmission before execution of interruption programs. In this sense, there is a wide ignorance about the prevalence of people in chronic phases that may represent a financial cost for the health system in the future if diagnostic and treatment activities are not carried out [15].

Due to advance missionary work throughout the national territory, patrolling activities, public order maintaining, protecting sovereignty in border areas and training for combat, the operational personnel of the Colombian National Army is exposed to different transmission cycles of the parasite mediated mainly by non-domiciliated triatomines. Furthermore, the Colombian National Army is an institution made up of Colombians from different urban and rural geographic areas of the country; therefore it grouped a large number of young adults who lived in areas with transmission before the implementation of interruption of transmission programs. According to the reports of Sistema Nacional de Vigilancia (SIVIGILA), during the years 2015 to 2018 have been reported 40 cases of infection with *T*. *cruzi* in active members of the Colombian Army. However, dimension of the infection is unknown inside the institution. The main aim of this study was to evaluate the prevalence of chronic phase infection with *T*. *cruzi* in active military population of the Colombian National Army gathered in five departments during 2018, as an initial part of surveillance and control program of the Chagas disease inside the military forces of Colombia.

## Materials and methods

### Study area

**Fig 1:**
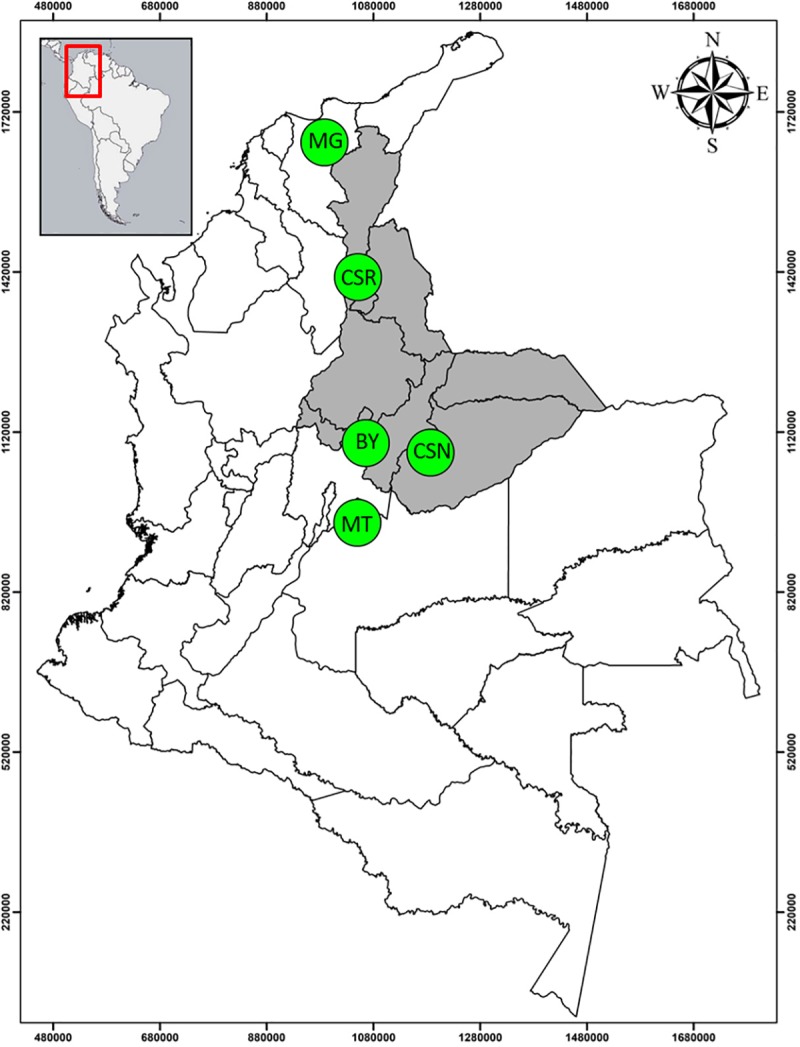
Sampling map in active military population of The Colombian National Army gathered in five departments during 2018. A total of 295 individuals were sampled in five BITER as described below: Boyacá or BY (N = 46), Casanare or CSN (N = 66), Cesar or CSR (N = 65), Magdalena or MG (N = 55) & Meta or MT (N = 63). In gray are highlighting the most endemic areas for Chagas disease according to The National Institute of Health in Colombia. These endemic areas were used as inclusion criteria for sampling the military personnel in this study.

Due to the high flow of military population between operational areas that were included in this study, the samples collection was carried out during the first six months of 2018in five training and re-training battalions (BITER), located in the departments of Boyacá (5°29′31″N 73°29′12″W), Casanare (5°19′50″N 72°23′26″W), Cesar (8°18′24″N 73°36′55″W), Magdalena (10°35′28″N 74°11′06″W) and Meta (4°08′33″N 73°37′46″W) (Fig 1). These BITER meet different units of the National Army gathered in each department for training and re-training actions during specific times of the year.

### Study design

A descriptive cross-sectional study was carried out according to the following inclusion criteria as military population over18 years old and minimum 3 months patrolling in endemic areas of the Chagas disease. In contrast, previous diagnosis of Chagas disease was taken as exclusion criteria. Samples were taken between January and June of 2018. For serological study, a sample of individuals of proportional size was calculated for each department that was done spontaneously in accordance to the arrival of different military units to the BITER.

### Sample size

In order to estimate the sample size, we accessed the database of the Ministry of National Defense and Management of Military Health of Colombia, which contains the information of the active military population gathered in five departments during 2018. Sample size (N) was calculated using the Epi-info V 2000 software with the priors: 5% confidence level, infection frequency of 3% [21] and error of 2%. A sample size of 280 people was estimated; however it was increased by 5% due to high flow of military population between operational areas. The sample was done for convenience and proportionally distributed in each department as follows: Boyacá (16%), Casanare (22%), Cesar (22%), Magdalena (19%) and Meta (21%).

### Sample collection

Approximately 5 mL of whole blood was collected by venipuncture using needles and vacutainer tubes. Each sample was divided for serological and molecular tests. An aliquot of 1000μL was mixed with an equal volume of Guanidine buffer (GBE) for molecular diagnostics. In order to obtain serum, 4mL of the sample was centrifuged for 15 minutes at 1000g, then we placed the supernatant into a new 1.5mL tube which were stored at -20° C until processing.

### Serological and molecular tests

All the patients were evaluated by two Enzyme Linked ImmunoSorbent Assay (ELISA) tests with different principles, according to the recommendations made by suggestions of National Institute of Health, Colombia. All the samples were subjected to a commercial ELISA of total antigens (ELISA Chagas III Bios Chile) and analyzed with the ELISA test with recombinant antigens (DiaPro Diagnostic Bioprobes *T*. *cruzi*-AB), following the manufacturer's instructions. The incongruent samples were analyzed by two additional serological tests: Indirect Immunofluorescence Assay (IFA) and Hemagglutination Inhibition test (HAI). The incongruent samples; reactive to at least one of two complementary tests were considered positive. The detailed protocols for all serological tests can be found at protocols.io: https://www.protocols.io/view/protocol-for-detect-trypanosoma-cruzi-by-indirect-62khgcw. Moreover, total DNA was extracted from the seropositive samples stored in GBE using Invisorb^®^ Spin Universal Kit of STRATEC Molecular GmbH. The absence of inhibitors was evaluated by amplifying the *CytB* gene using the forward 5'-CCCCTCAGAATGATATTTGTCCTCA-3' and reverse 5'- CCATCCAACATCTCAGCATGATGAAA-3 primers following thermal conditions reported by [22]. For the detection of *T*. *cruzi*, satellite DNA amplification of 188bp was done using the TcZ1 (5'-GCT CTT GCC CAC AMG GGT GC-3 ') and TcZ2 (5'-CAA GCA GCG GAT AGT TCA GG-3'). PCR was performed in a final volume of 20 μL of reaction containing 5 μL of DNA from blood treated with guanidine hydrochloride, 3mM MgCl_2_, 1X Buffer, 0.8μM of each primer, 0.05mM of dNTPs, 0.05U/μL of Taq DNA polymerase Immolase DNA Pol BIOLINE and 8.3μL of ultrapure di-destilated water. The amplification cycles were carried out in a CFX-1000 touch BIORAD thermal cycler with an initial temperature of 94°C for 3 minutes, followed by 40 cycles of denaturation at 94°C for 45 seconds, annealing at 68°C for one minute and elongation at 72°C for 1 minute and a final extension of 72°C for 10 minutes according to the conditions reported by [23].

### Analytical approach

All the participants who made part in the serological study were asked a clinical-epidemiological survey that contained personal information such as: age, sex, service time in the Army, patrolling department with high transmission and place of birth. Regarding the place of birth, two groups were assigned: (i) population with potential transmission of Chagas disease located in municipalities at a height below 2000mamsl with previous triatomine reports. (ii) Population without potential transmission located at more than 2000mamsl or located below this altitude without triatomine reports [3, 5]. Further, some epidemiological relevance data was asked, such as recognition of triatomines, for which dissected samples of the main vectors of Colombia were showed; places where triatomines sighting before and during the service, family history of Chagas disease, blood transfusion and previous infection with Leishmaniasis. All the variables were subjected to a descriptive analysis based on frequencies and percentages for qualitative variables.

### Ethics statement

This study was approved by the Ethics Committee of the Central Military Hospital (HMC) of Bogotá, Colombia, by official approval of December 13, 2017. All participants accepted their voluntary participation by signing an informed consent also endorsed by the HMC. All participants received the results of serological tests in a confidential manner and the participants with positive results were notified to the military health unit for management and delivery of treatment.

## Results

A total of 295 active members of the Colombian National Army participated in the study. All participants were male with an age range between 21–46 years, and average of age of 31 (SD = 5,96) years old. 194 (66%) of the military population included were soldiers, 66 (30%) sub-officers and 13 (4%) officers. Finally, 208 (70.5%) of the participants were born and lived the first years of their childhood in municipalities with potential transmission of *T*. *cruzi* (Table 1).

**Table 1:** Sociodemographic and epidemiological variables in the study population (N = 295) for prevalence of *Trypanosoma cruzi* infection.

Variable	N (%)
Age	
20–24 25–29 30–34 35–39 40–44 >45 Without Data	51 (17.3%)
	60 (20.3%)
	89 (30.2%)
	77 (26.1%)
	12 (4.1%)
	3 (1%)
	3 (1%)
Military rank	
Officers Sub-officers Soldiers	13 (4%)
	88 (30%)
	194 (66%)
Birth in municipalities with potential transmission of *T*. *cruzi*	
Yes No	208 (70.5%)
	87(29.5%)

71% of the participants clearly recognized the triatomines. 71% of this group said they had seen or had contact with triatomines during tasks of service. Finally, 3% of participants have close relatives with *T*. *cruzi* infection (Table 2).

**Table 2:** Knowledge about Chagas disease according to clinical and epidemiological survey.

Variable	N (%)
Recognition of triatomines	
Yes	209 (71)
No	83 (28)
N/A	3 (1)
In which place have you sighting triatomines?	
Before service	20 (9)
During service (operational zones)	153 (73)
Before and during service	21 (10)
N/A	15 (8)
Blood transfusion	
Yes	7 (2.3)
No	284 (96.3)
N/A	4 (1.4)
Family history of infection with *T*. *cruzi*	
Yes	9 (3)
No	278 (94.3)
N/A	8 (2.7)
Previous Leishmaniasis	
Yes	77(26.1)
No	218 (73.9)

Serological tests were performed on the 295 samples collected. Two samples were positive by applying two ELISA tests, for an infection prevalence of 0.68% (95% CI: 0.22–1.16). Two additional samples had incongruent results between two ELISA tests, however the IIF and IHA test showed non-reactive results. The molecular diagnosis of blood samples stored in BCG (Guanidine Chloride Buffer), by amplifying the satellite DNA of *T*. *cruzi*, did not show the presence of parasites in seropositive individuals.

The two seropositive members of the National Army that were not symptomatic correspond to two professional soldiers born in municipalities with a tradition by domiciled vectors in the departments of Casanare and Boyacá. Furthermore, one of the seropositive patients has a brother with a history of infection with *T*. *cruzi*.

## Discussion

The present study, is the first report of the prevalence of *T*. *cruzi* infection in active military population of The Colombian National Army gathered in five departments during 2018.The serological study of *T*. *cruzi* infection in specific population groups and young groups is one of the measures used to assess infection intensity or to check the interruption of transmission after development of intervention and vectorial control programs [24]. Even though, the evaluation of the adult population infection is considered uninformative in these scenarios, its utility is high for prevention programs of vectorial or oral transmission as consequence of work activities, since it allows the promotion of self-care, the identification of cases, treatment supply that prevents the heart failure and the reduction of disease burden[15].

A prevalence of *T*. *cruzi* infection of 0.68% (95% CI: 0.22–1.16) is reported for military population of The Colombian National Army gathered in five departments during 2018with traditional Chagas disease transmission. Our value of prevalence found here is slightly lower than estimated for the civil population of the country that lives in areas with triatomine presence, according to meta-analyzes results recently published [15, 16]. In Colombia, comparisons in the prevalence of *T*. *cruzi* infections in different age groups, suggest that the greatest trouble is related to adults where about 3% (95% CI, 1.0–5.0) is infected with the parasite [15, 16]. This position suggests the need to include an adult serological screening that allows both the identification of asymptomatic patients who acquired the infection before consolidation of the National Transmission Interruption Program, as well as, cases that can be acquired during operational activities in areas with presence of vectors [10, 20, 25]. The identification of these cases could provide to the prevention of cardiac failures belong to the chronic phase, throughout chemotherapeutic management.

Serological studies accomplished on adult population in the departments of Antioquia and Cesar showed values of seroprevalence lower than found it in this study [26, 27]. This may be related to the fact that blood banks are located in urban centers where they carry out donor recruitment [26]. Otherwise, the collections in blood banks fulfill with some selection criteria aimed at minimizing people participation that were born in endemic areas [24, 26]. A different plight occurred in this study, where 70.5% of the participants were born in municipalities with reports of infected triatomines [3, 8]. Moreover, 3% of the participants reported a family medical history of Chagas disease. These situations simultaneously with the negative results of the molecular diagnosis, suggest that the infection of the two professional soldiers could occur in early stages of their life and municipalities of birth, which were widely known for the presence of domiciled *R*. *prolixus*.

Studies on the prevalence of *T*. *cruzi* infection in an active military population are not a frequent activity in military health programs in Colombia as neither in other countries of the region. This may be related to the low occurrence of cases that arise for this event in comparison with other vector borne diseases (VBD) such as leishmaniasis, malaria and dengue [28]. However, the report of an outbreak related to oral transmission of Chagas disease in the Catatumbo region, where 17% (25/144) were infected with *T*. *cruzi*, confirms the risk to which this population is exposed during their missionary activities [13, 29]. In this sense, the serological analysis of the military population exposed to the *T*. *cruzi* transmission during training or patrolling in endemic areas, has been proposed as an activity that allows improving the surveillance strategies that protect humans and canines belonging to military forces in endemic areas of *T*. *cruzi*[30, 31].

Chagas disease has a complexity of diagnostic process, which is hampered by the lack of a gold standard, availability of multiple types of assays with varying sensitivity and specificity, and the sheer difficulty of detecting the parasite in the chronic phase leaves the diagnosis to be only conducted for this phase through out detection on antibodies [32]. Moreover, another of the common problems in serological diagnosis of Chagas disease is the cross-reactions that result from infections with another type of trypanosomatids [33–35]. The phylogenetic proximity between *Leishmania spp* and *T*. *cruzi* is observed in shared antigens that cause troubles of sensitivity and specificity of serological tests [36–37]. Cutaneous leishmaniasis caused by *L*. *braziliensi*s and *L*. *panamensis* is the VBD with the highest prevalence and incidence on members of the Colombian National Army [38]. In the present study, 26.1% of the participants reported previous infection with Leishmaniasis. However, the ELISA tests used for this study did not cross-react with people with a medical history of Leishmaniasis. Due to the previous information, ELISA tests can continue to be used in order to estimate the prevalence of *T*. *cruzi* infection in military population located in other endemic areas for Chagas disease in Colombia.

71% of studied population correctly identified the triatomines and their ecological role in the epidemiology of Chagas disease. These results are much higher than those reported in other endemic areas of the country [39, 40]. Former data can be the result of the actions in concordance with prevention and control programs of VBD within the military forces, which has the function of training in recognition and adoption of protection measures such as the use of repellents in the area of exposed skin, as well as, the use of insecticides to impregnate uniforms. Actually, the existence of this program seems to have great impact in the operation areas, it is necessary to characterize the presence of risk of vector and oral transmission within battalions and other units established in endemic areas that gather the population in different seasons [31].

Our study has certain limitations. The development of researches in military population in operational areas turns out to be complex due to high mobility, staff rotation and the mission accomplished by the crew in the area. This situation creates difficulties for the collection of samples since all the members are not gathered in a specific place, therefore, our results cannot be generalized to the entire military population of Colombia. Additionally, for the recognition of vectors, we teach dissected images and triatomines to the interviewees, it is possible that the group of participants who said they had contact with triatomines during the presentation of the service, confused these vectors with other reduced predators and phytophagous who inhabits in colombian jungles. Finally, another important limitation of our study is the impossibility of evaluating the impact of cross-reactions or co-infection with *Trypanosoma rangeli*, another trypanosomatide that circulates in Colombia due to the absence of reference serum for this parasite [32, 41]

In conclusion, our study reports for the first time the *T*. *cruzi* infection in an active military population of the Colombian National Army. Even though, the methodology used is not possible to extend to the entire population that makes up the institution, this allows estimating the infection dimension in the members gathered in Boyacá, Cesar, Santander, Casanare and Arauca departments during the year 2018. Our results of serological and molecular diagnosis suggest the lack of recent infection, which could be attributed to the instructions and training from the prevention program within the Army. However, these results show the presence of a high number of people who were born and lived during their first years in areas with presence of domiciled vectors, before the establishment of the interruption programs by chemical control. This situation, added to the continuous exposure of active military personnel when carrying out actions of patrolling, territorial control, maintenance of public order and protection of sovereignty, justifies the continuity and maintenance of disease screening.
